# Abnormally Expressed lncRNAs as Potential Biomarkers for Gastric Cancer Risk: A Diagnostic Meta-Bioinformatics Analysis

**DOI:** 10.1155/2022/6712625

**Published:** 2022-11-04

**Authors:** Ying-ying Dong, Quan Zhou, Hao Li, Zhi Lv, Yuan Yuan, Li-ping Sun

**Affiliations:** ^1^Tumor Etiology and Screening Department of Cancer Institute, And Key Laboratory of Cancer Etiology and Prevention in Liaoning Education Department, The First Hospital of China Medical University, Shenyang 110001, China; ^2^Key Laboratory of GI Cancer Etiology and Prevention in Liaoning Province, The First Hospital of China Medical University, Shenyang 110001, China; ^3^Department of Clinical Laboratory, The First Hospital of China Medical University, Shenyang 110001, China

## Abstract

**Background and Aims:**

Abnormal expression of lncRNAs is relevant to the occurrence and development of gastric cancer (GC), but the significance remains inconclusive. We performed a diagnostic meta-bioinformatics analysis to elucidate the association between lncRNA expression and GC risk.

**Methods:**

Published datasets were selected from PubMed, Embase, CNKI, and Web of Science, up to 1st December 2021. The pooled sensitivity (SEN), specificity (SPE), positive likelihood ratio (PLR), negative likelihood ratio (NLR), diagnostic odds ratio (DOR), and area under the curve (AUC) were calculated to evaluate the diagnostic value. RNA sequencing data were downloaded for validation.

**Results:**

54 studies with 4671 patients and 4652 matched controls were included in the meta-analysis. The pooled SEN, SPE, PLR, NLR, DOR, and AUC were 0.71, 0.76, 2.9, 0.39, 8, and 0.79, respectively. Subgroup analyses showed that the DOR and AUC of intergenic lncRNAs, circulating lncRNAs, larger sample size (>200), and high-quality (NOS score ≥ 7) groups were superior to antisense lncRNAs, tissue lncRNAs, smaller sample size (≤200), and low-quality (NOS score < 7) groups, respectively. However, only circulating lncRNAs had significantly higher diagnostic utility than that tissue lncRNAs. Nine differentially expressed lncRNAs in the meta-analysis were verified in TCGA-STAD. PVT1 was the most effective single lncRNA, with AUC of 0.949, SEN of 0.808, and SPE of 0.969, while PVT1 and C5orf66-AS1 were the most effective combination, with AUC of 0.972, SEN of 0.941, and SPE of 0.937.

**Conclusion:**

Abnormally expressed lncRNAs, especially circulating lncRNAs, might be potential diagnostic biomarkers for GC risk. A novel combined model of lncRNAs might achieve better GC diagnosis performance.

## 1. Background

Long noncoding RNAs (lncRNAs) regulate cell proliferation, apoptosis, differentiation, and metastasis, which all are associated with multiple diseases, including tumorigenesis [1]. In tumorigenesis, lncRNAs are involved at the transcriptional, posttranscriptional, and epigenetic levels. Based on their genomic position in relation to the protein-encoding gene, lncRNAs can be divided into sense, antisense, bidirectional, intergenic, and intronic lncRNAs [2]. The location of the lncRNA directly influences its function in the genome. Intergenic lncRNAs regulate the expression of upstream and downstream genes, while antisense lncRNAs bind to mRNA of complementary genes to protect mRNA from RNase-mediated degradation [3]. Many studies have reported that tissue or blood lncRNAs can be used as biomarkers for cancer diagnosis. lncRNAs show broad prospects as molecular biomarkers because of their specific expression and regulation dissimilarity in specific cancers. For example, lncRNA prostate cancer-associated 3 (PCA3) is used in the diagnosis of prostate cancer [4], and highly upregulated in liver cancer (HULC) is meaningful to the diagnosis of liver cancer and the identification of hepatic metastasis in colorectal cancer [5].

Gastric cancer (GC) is the top 5 cancer type and is the fourth leading cause of cancer-related death worldwide according to Global Cancer Statistics 2020 [6]. Approximately half of those GC cases come from East Asia, especially China [7]. Prevention and early diagnosis of GC are essential. Since a positive association between H19 overexpression and GC was reported by Wu et al. in 1997 [8], accumulating studies have focused on the relationship between lncRNA expression and GC risk. Owing to the dysregulated expression levels, lncRNAs have been classified as oncogenic molecules and tumor suppressors. Some studies have reported that HOX transcript antisense RNA (HOTAIR) [9], LINC00152 [10], and LIFR-AS1 [11] were upexpressed in GC tissue, while C5orf66-AS1 [12] and lnc-GNAQ-6:1 [13] were downexpressed in GC serum, and the exosomal lnc-GNAQ-6:1 exhibited a more favored ROC than traditional biomarkers such as serum carcinoembryonic antigen (CEA), cancer antigen 19-9 (CA19-9), and carbohydrate antigen 72-4 (CA72-4) [13]. However, some studies have shown inconsistent results, which confuses us about the value of lncRNA expression in GC risk assessment. For instance, Fei et al. reported that LINC00982 was upexpressed in GC tissue [14], but Zheng et al. found that it was low expressed and acted as a tumor suppressor, and its overexpression would impair the proliferative, migratory, and invasive properties of GC cells [15]. So far, two meta-analyses investigated the diagnostic accuracy of diverse lncRNAs in GC patients [16, 17], one of which mentioned a stratified analysis of tissue and plasma samples. However, other aspects of lncRNA biology, including the impact of lncRNA genomic location on its diagnostic value, have not been explored. Moreover, only a few lncRNAs have been confirmed to have diagnostic efficacy, and the diagnostic SEN and SPE of a single lncRNA are generally low.

Therefore, we conducted a diagnostic meta-analysis exploring the association between lncRNA expression and GC risk, taking genome location and sample source into account. Additionally, using TCGA database, we constructed individual and combined lncRNA models of GC risk assessment for bioinformatics analysis and validation.

## 2. Materials and Methods

This systematic review meta-analysis was conducted in accordance with the Preferred Reporting Items for Systematic Reviews and Meta-Analyses (PRISMA) guidelines [18, 19].

### 2.1. Publication Search Strategy

We systematically searched PubMed, Embase, CNKI, and Web of Science for studies focusing on the relationship between lncRNA expression and GC. Our medical subject heading terms (for PubMed), EMTREE terms (for Embase), and text (for others) were “(RNA, Long Non-coding OR long untranslated RNA OR long non-coding RNA OR lncRNA) AND (Stomach Neoplasms OR stomach cancer OR gastric cancer).” We searched the databases of each primary study up to December 1, 2021.

Eligible studies met the following criteria: (1) studies that reported lncRNA expression data from patients were identified as GC by postoperative pathologic check according to the guideline of the National Comprehensive Cancer Network (NCCN), the European Society for Medical Oncology (ESMO), and the Chinese Society of Clinical Oncology (CSCO); (2) studies provided sufficient data to evaluate the relationship between lncRNA expression and the diagnosis of GC versus different control types (adjacent nontumor tissue, benign gastric lesions, or healthy volunteers); (3) datasets generated using qRT-PCR; (4) GAPDH and *β*-actin were used as qRT-PCR reference genes; and (5) the studies provided sufficient information to construct a 2 × 2 contingency table, with false/true positives/negatives provided. Studies were excluded if (1) the studies or data were duplicated, letters to the editor, commentaries, and review papers; (2) they were not relevant to GC diagnosis/risk or lncRNA expression; and (3) contained a lack of variable data or tables [20].

### 2.2. Data Extraction

Two investigators (Yingying Dong and Quan Zhou) extracted all the data independently and reached a consensus regarding all items. Controversial sections were verified and resolved by Dr. Liping Sun. The following is the information extracted from the included literature: the author's name, year of publication, country of origin, number and source of cases and controls, differential lncRNA expression, area under the curve (AUC) of the summary receiver-operating characteristic (SROC) curve, cut-off, sensitivity (SEN) and specificity (SPE), positive likelihood ratio, and negative likelihood ratio.

### 2.3. Methodologic Quality Assessment

Yingying Dong and Quan Zhou assessed the data quality using the Newcastle–Ottawa quality scale (NOS). A form which comprised three parts was used to assess the quality of nonrandomized studies in meta-analyses: (1) the selection of study groups, (2) the comparability of study groups, and (3) the assessment of exposure or outcomes. Each study was assigned a score of 0–9, with a score of at least 7 (of 9) indicating high quality.

### 2.4. Public Data Processing and Tools

lncRNA classification was based on the latest nomenclature outlined on the HGNC website (http://www.genenames.org/). Genomic positions of lncRNA in relation to protein-encoding genes were identified using the UCSC database (http://genome.ucsc.edu/) and LNCipedia version 5.2 (https://lncipedia.org/). A total of 407 GC samples from TCGA project (https://cancergenome.nih.gov/), including 375 cancer cases and 32 cancer cases with adjacent nontumor tissue (ANT), were downloaded. Morpheus database (https://software.broadinstitute.org/morpheus/) was used to identify genes that were differentially expressed in GC. RNA-seq raw read counts were converted to transcripts per million (TPM) values to normalize all samples.

### 2.5. Statistical Analysis

STATA 15.0 (Stata Corporation, College Station, TX, USA), Meta-Disc 1.4 (XI Cochrane Colloquium, Barcelona, Spain), RevMan 5.3 (The Nordic Cochrane Centre, The Cochrane Collaboration, Copenhagen), SPSS 21.0 (IBM Corp., Armonk, NY, USA), and GraphPad Prism 7.0 software (GraphPad Software Inc., La Jolla, CA, USA) were used for statistical analysis.

The Spearman correlation coefficient, Cochran's *Q* test, and inconsistency index (*I*^2^) test were used to confirm the heterogeneity of threshold or nonthreshold effects. If there was heterogeneity (*I*^2^ ≥ 50% or *P* ≤ 0.05), the random effects model was adopted. If there was no heterogeneity, the fixed effects model was used. The subgroup differences of AUC were conducted by using a two-sided *Z*-test at a significance level of 0.05. Sensitivity analysis was performed by removing each study from the analysis to determine its impact on the overall effect. Metaregression was performed to find the origin of heterogeneity. Pooled SEN, SPE, diagnostic odds ratio (DOR), positive likelihood ratio (PLR), and negative likelihood ratio (NLR) values were generated using bivariate analysis. Deeks' funnel plots and symmetry tests were used to investigate publication bias, with the significance threshold set at *P* < 0.01. lncRNA expression differences were analyzed by the Mann-Whitney *U* test. Binary logistic regression analysis (enter method) was used to construct the combined diagnostic model. AUC was used to evaluate diagnostic efficacy. *Z*-test was conducted to determine the difference of AUC between different GC stages. *P* < 0.05 was considered statistically significant.

(1) $$\begin{array}{c} Z=\frac{\text{AUC}1-\text{AUC}2}{\sqrt{SE1^{2}+SE1^{2}}}. \end{array}$$

## 3. Results

### 3.1. Literature Search and Study Characteristics

**Figure 1:**
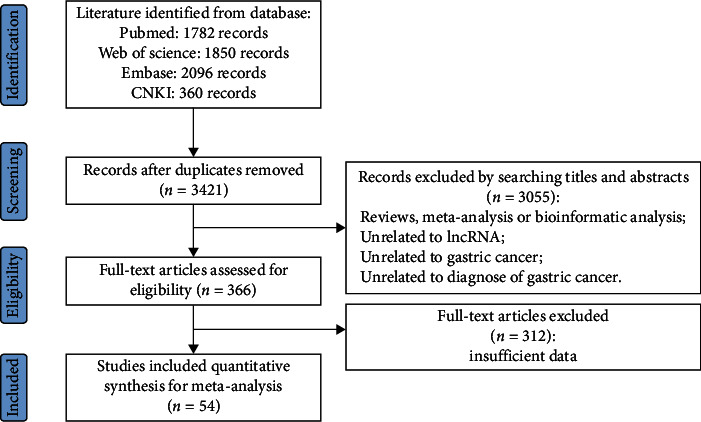
Process of study selection.

The study selection process is shown in Figure 1. Firstly, we retrieved 6088 articles from all selected databases; then, we excluded 2667 duplicates. After reviewing the titles and abstracts, 3055 publications were found to be irrelevant. After a full-text review, 54 studies remained to be analyzed. The diagnostic accuracy was reported separately for different lncRNAs or different sample types, so the reported data from 4671 patients and 4652 matched controls were analyzed. The main study characteristics are shown in Table 1. Sample types included tissue [9, 11, 21–48], circulating (plasma and serum) [12, 13, 47, 49–65], and gastric juice [21, 64, 66]. Most studies took specimens from Chinese population, and six studies took samples from Japanese [49], Egyptian, [53], or Iranian populations [31, 32, 34, 37].

We assessed the quality of included studies using NOS and found that the quality of the enrolled studies was acceptable. Thirty-eight studies [9, 12, 14, 21–27, 29–31, 33, 34, 36, 38–40, 42, 46–50, 52–55, 57, 62, 63, 65, 66] were of high quality while the other 16 studies [28, 32, 35, 37, 41, 43, 47, 51, 56, 58–61, 64, 67] were of moderate quality.

### 3.2. Determination of Diagnostic Performance

**Figure 2:**
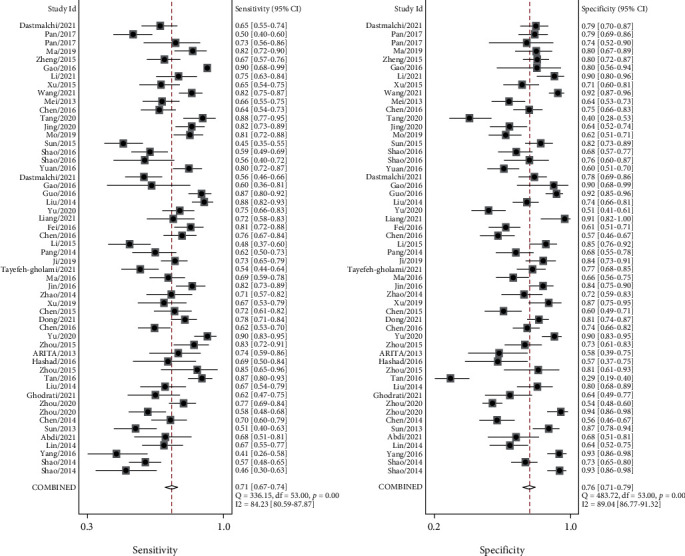
SEN and SPE of lncRNA assay for diagnosis of GC. The pooled SEN: 0.71 (95% CI: 0.67-0.74); the pooled SPE: 0.76 (95% CI: 0.71-0.79).

**Figure 3:**
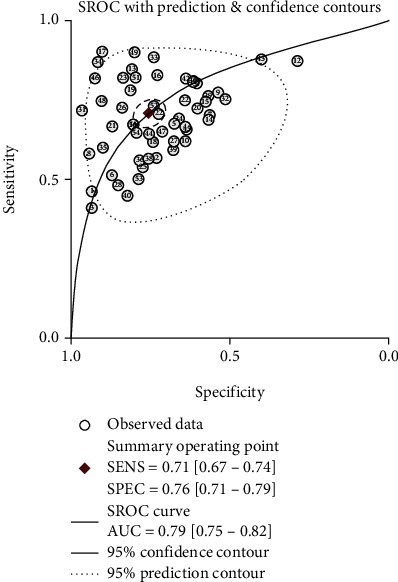
SROC for lncRNA expression in GC diagnosis. One cycle represents an individual study. The AUC is 0.79.

Because significant heterogeneity was observed between studies for the high *I*^2^ values in SEN (84.23%, *P* < 0.001), SPE (89.04%, *P* < 0.001), PLR (83.95%, *P* < 0.001), NLR (80.61%, *P* < 0.001), and DOR (78.9%, *P* < 0.001) (Table 2), we choose the random effects model for further analysis. Forest plots of the pooled SEN and SPE for lncRNAs as biomarkers are shown in Figure 2. The pooled SEN for the data was 0.71 (95% CI: 0.67–0.74), and the pooled SPE was 0.76 (95% CI: 0.71–0.79). The PLR, NLR, and DOR were 2.9 (95% CI: 2.5–3.4), 0.39 (95% CI: 0.34–0.43), and 8 (95% CI: 6–10), respectively (Table 2 and Figure 2). The AUC was 0.79 (95% CI: 0.75–0.82; Figure 3), indicative of being a suitable diagnostic index (Table 2).

### 3.3. Study Heterogeneity

In order to determine the potential source of heterogeneity, we performed subsequent analysis on the threshold effect and nonthreshold effect. Spearman's rank correlation was used to assess the heterogeneity of the threshold effect since Spearman's coefficient was 0.25 (*P* = 0.069). There was no heterogeneity from the threshold effect. In addition, the Cochran *Q* of DOR is commonly used to detect nonthreshold effect heterogeneity; we analyzed heterogeneity with Cochran's *Q* test and *I*^2^ test and found that their DOR values were 251.75 (*P* < 0.001) and 78.9% (supplement Table S1), indicating that there was considerable heterogeneity caused by nonthreshold effect. Then, we further performed a series of analyses to find the sources of heterogeneity.

### 3.4. Subgroup Analysis and Metaregression

We divided the 54 studies into four subgroups for stratified analyses, including the genomic location of the lncRNA (intergenic vs. antisense), sample type (circulating vs. tissue), sample size (≤200 vs. > 200), and quality (NOS score < 7 vs. ≥7). The details are shown in Table 2. In the location subgroups, the diagnostic SEN of lncRNAs extracted from intergenic was 0.72 (95% CI: 0.66-0.77), and the SPE was 0.78 (95% CI: 0.72-0.83), with the pooled DOR of 9 (95% CI: 6-13) and AUC of 0.81 (95% CI: 0.78-0.84). The pooled SEN and SPE of lncRNAs of antisense were 0.73 (95% CI: 0.66-0.79) and 0.71 (95% CI: 0.61-0.80), with DOR of 7 (95% CI: 4-10) and AUC of 0.78 (95% CI: 0.75-0.82). From the perspective of sample type, the diagnostic accuracy of the circulating group was significantly higher than that of the issue group, with the SEN increasing from 0.69 (95% CI: 0.65-0.73) to 0.76 (95% CI: 0.71-0.81) and the SPE increasing from 0.72 (95% CI: 0.68-0.76) to 0.79 (95% CI: 0.71-0.86). The DOR increased from 6 (95% CI: 5–7) to 12 (95% CI: 7–20), and the AUC increased from 0.77 (95% CI: 0.73–0.80) to 0.84 (95% CI: 0.80–0.87), SEN 0.76 (95% CI: 0.71–0.81) vs. 0.69 (95% CI: 0.65–0.73), SPE 0.79 (95% CI: 0.71–0.86) vs. 0.72 (95% CI: 0.68–0.76), PLR 3.7 (95% CI: 2.6–5.3) vs. 2.5 (95% CI: 2.2–2.9), NLR 0.3 (95% CI: 0.24–0.37) vs. 0.42 (95% CI: 0.38–0.48), DOR 12 (95% CI: 7–20) vs. 6 (95% CI: 5–7), and AUC 0.84 (95% CI: 0.80–0.87) vs. 0.77 (95% CI: 0.73–0.80), respectively.

Compared to the groups of sample size ≤ 200, the diagnostic value of the groups with sample > 200 demonstrated better, SEN 0.75 (95% CI: 0.70–0.80) vs. 0.68 (95% CI: 0.63–0.72), SPE 0.77 (95% CI: 0.71–0.82) vs. 0.75 (95% CI: 0.69–0.79), PLR 3.2 (95% CI: 2.5–4.2) vs. 2.7 (95% CI: 2.2–3.2), NLR 0.33 (95% CI: 0.26–0.41) vs. 0.43 (95% CI: 0.39–0.48), DOR 10 (95% CI: 6–15) vs. 6 (95% CI: 5–8), and AUC 0.82 (95% CI: 0.79–0.86) vs. 0.76 (95% CI: 0.72–0.80), respectively. In terms of the study quality, the studies of NOS score ≥ 7 had a little higher diagnostic value than the studies of NOS score < 7, SEN 0.71 (95% CI: 0.66–0.75) vs. 0.71 (95% CI: 0.65–0.77), SPE 0.76 (95% CI: 0.72–0.80) vs. 0.75 (95% CI: 0.64–0.83), PLR 3.0 (95% CI: 2.5–3.5) vs. 2.8 (95% CI: 2.0–4.0), NLR 0.39 (95% CI: 0.34–0.44) vs. 0.38 (95% CI: 0.31–0.47), DOR 8 (95% CI: 6–10) vs. 7 (95% CI: 5–12), and AUC 0.80 (95% CI: 0.76–0.83) vs. 0.78 (95% CI: 0.74–0.82), respectively. Additionally, we also detected the heterogeneity from subgroups; the *I*^2^ of variates such as lncRNAs extracted from tissue and sample size ≤ 200 decreased obviously from the different groups, which suggested that these variables may be the sources of potential heterogeneity (Table 2).

Then, we constructed a metaregression in terms of the specified covariates including location, sample type, sample size, and quality (Table 3, A–E). During metaregression, the covariate lacks a value, using 0 instead of it. According to the *P* value from large to small, “location,” “quality,” and “sample size” were eliminated one by one. The results showed that the significant heterogeneity of sample size groups and sample type groups was not affected by other covariables; this suggested that the sample type (RDOR = 1.82, 95% CI: 1.19-2.78, *P* = 0.0063) and sample size (RDOR = 1.71, 95% CI: 1.08-2.68, *P* = 0.0218) could be considered as the source of heterogeneity in the detection of gastric cancer.

### 3.5. Sensitivity Analysis and Publication Bias

By excluding individual studies, sensitivity analysis was used to test the impact on overall effects and changes in heterogeneity. As displayed in supplement Figure S1, none of the included individual studies were out of the upper or lower CI limits, suggesting that there was no single heterogeneity study with relatively large overall effects; the selected studies were homogeneously distributed.

**Figure 4:**
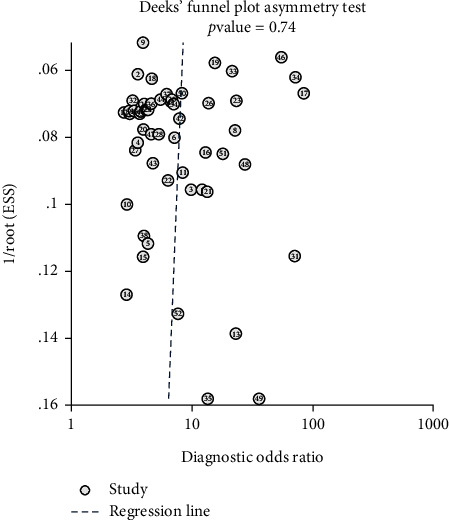
Funnel plot for the assessment of potential publication bias of the diagnostic studies. Each point represents a study, and the line is the regression line. The *P* value is 0.74, indicating that there was no publication bias.

No significant publication bias was found in this system. The slope coefficient did not indicate asymmetry, and the *P* value was 0.74 (Figure 4).

### 3.6. Clinical Utility of lncRNAs in the Diagnosis of GC

**Figure 5:**
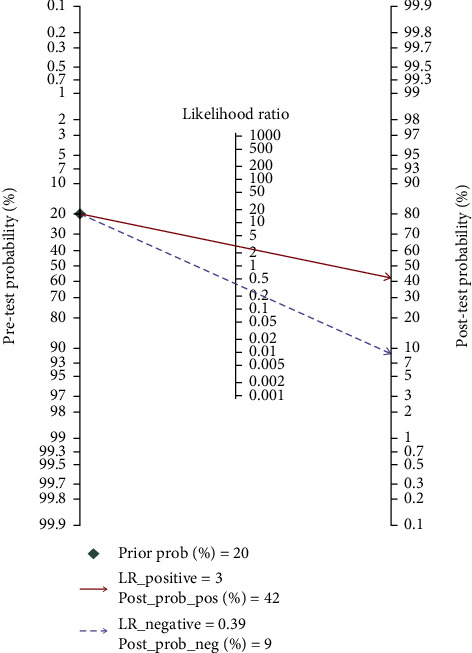
Fagan's nomogram of the lncRNA test for diagnosis of GC.

Fagan's nomogram was used to verify the probability of GC being detected by lncRNAs (Figure 5). For anyone having a pretest probability of 20%, if the lncRNA test in cancer detection is positive, the probability of GC after the test will increase to 42%. The negative result of lncRNA detection means that the probability of posttest in the same population will drop to 9%, suggesting that lncRNAs were a promising indicator for the diagnosis of GC.

### 3.7. Bioinformatics Verification of lncRNA Expression in GC

#### 3.7.1. Differential Expression of lncRNAs in TCGA-STAD Database

Using TCGA database, we verified expression differences of 37 lncRNAs derived from published data in GC. A total of 9 lncRNAs exhibited changed trends consistent with TCGA data, including AC064834.1, H19, HOTAIR, HULC, keratin 18 pseudogene 55 (KRT18P55), PVT1, urothelial cancer-associated 1 (UCA1), C5orf66-AS1, and LINC00086. However, the opposite was found in six lncRNAs, abhydrolase domain containing 11-antisense RNA1 (ABHD11-AS1), gastric cancer-associated transcript 2 (GACAT2), LINC00982, RP11-731F5.2, TINCR, and long intergenic nonprotein coding RNA, regulator of reprogramming (linc-ROR). In addition, for 8 upregulated lncRNAs in the published data, no significant difference was detected. We could not find any data for the remaining 14 lncRNAs in TCGA database (Table 4).

#### 3.7.2. The Diagnostic Efficacy of lncRNA Expression for GC in TCGA-STAD Database

**Figure 6:**
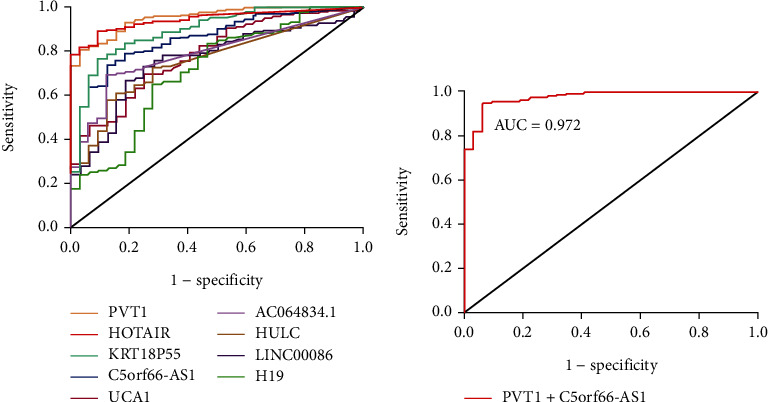
Diagnostic efficacy of single lncRNAs and combined model. (a) The ROC of 9 differentially expressed lncRNAs with the same trend in meta- and TCGA analysis for GC diagnosis. (b) The ROC of lncRNAs combined models: the AUC is 0.972.

The ROC of the above 9 differentially expressed lncRNAs for GC diagnosis in TCGA-STAD database is shown in Figure 6. PVT1 was a single lncRNA with the optimal diagnostic performance for GC, with an AUC of 0.949 (95% CI: 0.922–0.976), SEN of 0.808, and SPE of 0.969, while PVT1 and C5orf66-AS1 were the most effective combination, with an AUC of 0.972 (95% CI: 0.951–0.992), SEN of 0.941, and SPE of 0.937. The regression equation constructed by such two lncRNAs was logit(*P*) = −1.307 + 7.129 × PVT1 − 1.204 × C5orf66 − AS1 (Figure 6 and Table 5).

We further analyzed the diagnostic efficacy of this combined model for GC patients with different stages from TCGA database to fully reveal the dynamic changes. According to the *Z*-test, the diagnostic efficacy of stage I to stage IV gradually improved, and the AUC of stage IV was significantly higher than that of stage I (Table 6).

## 4. Discussion

Recent studies have assessed the utility of aberrant lncRNA expression profiles in differentiating between patients with GC patients and cancer-free individuals. However, the results of these studies are inconsistent. We performed this meta-analysis to evaluate whether, and which, lncRNAs have the potential to be biomarkers for GC diagnosis. In this study, we examined relevant articles published on 1st December 2021 and performed subgroup analysis based on the lncRNA genomic locations, sample source, sample size, and quality. We also conducted bioinformatics prediction analysis using TCGA data to further verify the meta-analysis results and construct a lncRNA model for GC diagnosis.

Our meta- and bioinformatics analysis showed that lncRNAs had better SPE (0.71), SEN (0.76), PLR (2.9), NLR (0.39), and AUC (0.79) for the diagnosis of GC than did certain protein markers. Many proteins, such as CEA and CA19-9, are used as biomarkers for GC diagnosis and have been used clinically [70]. However, lncRNAs act as precursor molecules, and their expression may be a better indicator of intrinsic tumor characteristics [71]. In general, the histological specificity of lncRNAs is superior to that of proteins [72], and lncRNAs have the potential advantage of being highly specific diagnostic biomarkers. Although HOTAIR is differentially expressed in various cancers, most lncRNA expression is histologically specific. For example, PCA3, PCGEM1, and PRNCR1 are highly expressed in prostate cancer, while differential HULU expression is related to liver cancer and liver metastasis [73].

When interpreting meta-analysis results, heterogeneity should be considered. The result of Spearman correlation analysis suggested there was no threshold effect. In addition, the *Q* test and the value of *I*^2^ > 50% indicated that there was heterogeneity of nonthreshold effect. However, sensitivity analysis found no obvious studies were identified as outlier studies. According to the subgroup analysis, the DOR and AUC of intergenic lncRNAs, circulating-based lncRNAs, larger sample size (>200), and high quality (NOS score ≥ 7) groups were superior to antisense lncRNAs, tissue-based lncRNAs, smaller sample size (≤200), and low quality (NOS score < 7) groups, respectively; however, only circulating lncRNAs had significantly higher AUC than that of tissue lncRNAs.

The genomic location of the lncRNA directly affects lncRNA function. However, in this study, no significant difference was found between the intergenic and antisense groups. The results of the subgroup analysis indicated that circulating group shares better performance than the tissue group; the SEN, SPE, PLR, NLR, and DOR in the blood samples were 0.76, 0.79, 3.7, 0.3, and 12, respectively. In the articles analyzed, the AUC of serum in the GC diagnosis using H19, HULC, and LINC01061 reached 0.943, 0.888, and 0.93, respectively. Future studies of lncRNA expression in the circulation of patients with early stage are necessary to identify a better diagnostic biomarker. The choice of the control group may explain some of the differences between the tissue and circulation groups. NAT was selected as a control for the tissue group, and healthy human serum was selected as a control for the serum group. However, NAT may be affected by the tumor microenvironment which may be why our results show that lncRNAs are not suitable for GC diagnosis from tissue samples. Besides, lncRNA encapsulated by exosomes is more stable in the serum and is not easily degraded by RNase. This suggests that serum-based detection of lncRNA expression is the preferred approach for future studies [49]. Of note, according to the results of subgroup analysis, more high-quality studies with a large sample size are required to further certify the diagnostic value of lncRNAs in GC. At the same time, metaregression analysis was implemented to explore the underlying causes of heterogeneity; we found that different lncRNA sample types and sample size might be the source of heterogeneity.

The reported lncRNA expression in GC most was corroborated by the results of TCGA database. We selected 9 lncRNAs with the same trend in meta- and TCGA analysis for subsequent diagnostic efficacy evaluation. Most previous studies have focused on single lncRNAs as potential biomarkers. However, several lncRNA combinations may have a better diagnostic performance [74]. Here, we identified a combined model of two lncRNAs (PVT1 and C5orf66-AS1) with an AUC of 0.972, which was higher than that of either PVTI (0.943) or C5orf66-AS1 (0.853) alone, indicating a more powerful ability to distinguish between patients with GC and healthy controls, especially for advanced GC patients. The stage often determines a patient's prognosis; early and advanced GC are treated differently. Surgery is often adopted in the early stages of GC, but in advanced stage cases, radiotherapy or chemotherapy is currently recommended for optimizing the chances of healing. Therefore, the prediction of GC staging is important. In this meta-analysis, the diagnostic efficiency of the lncRNA model in advanced GC was significantly higher than that in the early stage; therefore, we can assess tumor staging in a noninvasive manner, which may influence individual treatment planning. Increased lncRNA PVT1 expression could be a potential diagnostic biomarker for GC [75]. C5orf66-AS1 is an antisense lncRNA located in the first intron region of C5ORF66. C5orf66-AS1 overexpression promotes cervical cancer cell proliferation [76] and is associated with poor prognosis [77]. Previously, we showed that decreased serum levels of C5orf66-AS1 can be utilized for GC diagnosis, especially for early diagnosis [12]. Guo et al. found that abnormal hypermethylation around the C5orf66-AS1 transcription start site is related to its dysregulation and is tumor-specific [78]. It is expected that the combined model described in this study can be verified and applied to assess GC risk.

There are several limitations to be noted in the current meta-analysis: firstly, remarkable heterogeneity was observed in this study, although the results of subgroup analysis could explain some sources of heterogeneity. Secondly, due to a lack of sufficient sample size, we only found that circulating lncRNA was of higher diagnostic efficacy than tissue-based lncRNA, so the predictive ability between serum-based lncRNA and plasma-based lncRNA needs to be further studied. Finally, although we validated most of the results using bioinformatics analysis, the majority of patients included in our study were Chinese except for six studies; thus, the applicability to other races might be limited.

## 5. Conclusion

Together, these results provided evidence that abnormally expressed lncRNAs might be potential diagnostic biomarkers for GC diagnosis, especially circulating lncRNAs showed superior predictive ability, convenience, and feasibility. Furthermore, the novel combination model of PVT1 and C5orf66-AS1 might achieve better diagnostic efficacy and clinical potential in the prediction of GC. Due to the potential limitations, this study's clinical application warrants further investigation.

## Figures and Tables

**Table 1:** Study characteristics.

Author	Year	Ethnicity	Gene	Regulated	Location	Sample type	ROC	Cut-off	SEN	SPE	Total	Cancer	Control	Control sources	NOS	Reference
Shao et al.	2014	Chinese	AA174084	Down	Unknown	Gastric juices	0.848	0.88	0.46	0.93	130	39	91	NMMG+AG+GU	7	[21]
Shao et al.	2014	Chinese	AA174084	Down	Unknown	Tissue	0.676	11.62	0.57	0.73	268	134	134	ANT	8	[21]
Lin et al.	2014	Chinese	ABHD11-AS1	Up	Intergenic	Tissue	0.613	11	0.67	0.64	150	75	75	ANT	7	[22]
Yang et al.	2016	Chinese	ABHD11-AS1	Up	Intergenic	Gastric juices	0.653	6.53	0.41	0.934	130	39	91	NMMG+AG+GU	7	[66]
Abdi et al.	2021	Iranian	AC064834.1	Up	Unknown	Tissue	0.7	Unclear	0.675	0.675	80	40	40	ANT	7	[31]
Sun et al.	2013	Chinese	AC096655.1-002	Down	Intergenic	Tissue	0.731	13.955	0.513	0.872	156	78	78	ANT	8	[24]
Chen et al.	2014	Chinese	AC138128.1	Down	Unknown	Tissue	0.688	Unclear	0.7	0.56	188	94	94	ANT	7	[48]
Zhou et al.	2020	Chinese	BC002811	Up	Unknown	Serum	0.723	1.41	0.58	0.943	170	100	70	Benign gastric diseases	6	[60]
Zhou et al.	2020	Chinese	C5orf66-AS1	Down	Intergenic	Serum	0.688	0.134	0.775	0.536	419	141	278	GS+GA+GD	7	[12]
Roghieh et al.	2021	Iranian	DLEU1	Up	Antisense	Tissue	0.7	0.3357	0.62	0.646	100	50	50	ANT	6	[32]
Liu et al.	2014	Chinese	FER1L4	Down	Unknown	Tissue	0.778	15.43	0.672	0.803	122	61	61	ANT	8	[23]
Tan et al.	2016	Chinese	GACAT2	Up	Antisense	Plasma	0.622	6.625	0.872	0.282	197	117	80	Healthy blood	6	[61]
Arita et al.	2013	Japanese	H19	Up	Intergenic	Plasma	0.64	Unclear	0.74	0.58	76	43	33	Healthy blood	7	[49]
Zhou et al.	2015	Chinese	H19	Up	Intergenic	Plasma	0.838	Unclear	0.829	0.729	140	70	70	Healthy blood	7	[62]
Zhou et al.	2015	Chinese	H19	Up	Intergenic	Plasma (early cancer)	0.877	Unclear	0.855	0.801	52	26	26	Healthy blood	7	[62]
Chen et al.	2016	Chinese	H19	Up	Intergenic	Tissue	0.697	Unclear	0.62	0.74	256	128	128	ANT	7	[25]
Hashad et al.	2016	Egyptian	H19	Up	Intergenic	Plasma	0.724	0.5	0.6875	0.5667	62	32	30	Healthy blood	7	[53]
Yu et al.	2020	Chinese	H19	Up	Intergenic	Serum	0.943	Unclear	0.9007	0.9018	224	112	112	Healthy blood	7	[63]
Hui et al.	2021	Chinese	HEIH	Up	Intergenic	Tissue	0.784	Unclear	0.78	0.812	300	150	150	ANT	7	[33]
Sun et al.	2015	Chinese	HIF1A-AS2	Up	Antisense	Tissue	0.673	9.56	0.7229	0.6024	166	83	83	ANT	7	[39]
Xu et al.	2019	Chinese	HOTAIR	Up	Antisense	Tissue	0.8416	1.4	0.6667	0.8704	108	54	54	ANT	7	[9]
Zhao et al.	2014	Chinese	HULC	Up	Antisense	Tissue	0.769	10.88	0.707	0.724	116	58	58	ANT	7	[29]
Jin et al.	2016	Chinese	HULC	Up	Antisense	Serum	0.888	Unclear	0.82	0.836	210	100	110	Healthy blood	8	[55]
Ma et al.	2016	Chinese	KRT18P55	Up	Unknown	Tissue	0.733	Unclear	0.691	0.66	194	97	97	ANT	8	[27]
Samaneh et al.	2021	Iranian	KRT18P55	Up	Unknown	Tissue	0.6799	0.0023	0.5392	0.7745	204	102	102	ANT	7	[34]
Ji et al.	2019	Chinese	LINC00086	Down	Unknown	Plasma	0.86	0.3	0.726	0.838	242	168	74	Healthy blood	9	[54]
Pang et al.	2014	Chinese	LINC00152	Up	Intergenic	Tissue	0.645	4.385	0.625	0.681	142	71	71	ANT	6	[28]
Li et al.	2015	Chinese	LINC00152	Up	Intergenic	Plasma	0.657	Unclear	0.481	0.852	160	79	81	Healthy blood	7	[57]
Chen et al.	2016	Chinese	LINC00152	Up	Intergenic	Tissue	0.705	3.175	0.7629	0.567	194	97	97	ANT	7	[30]
Fei et al.	2016	Chinese	LINC00982	Up	Antisense	Tissue	0.742	Unclear	0.811	0.613	212	106	106	ANT	8	[14]
Liang et al.	2021	Chinese	LINC01061	Up	Intergenic	Serum	0.93	Unclear	0.717	0.965	82	53	29	Healthy blood	6	[58]
Yu et al.	2020	Chinese	linc-ROR	Down	Unknown	Tissue	0.6495	16.79	0.7524	0.5143	210	105	105	ANT	6	[35]
Liu et al.	2014	Chinese	ncRuPAR	Down	Intergenic	Tissue	0.84	4.97	0.8841	0.7391	276	138	138	ANT	7	[36]
Guo et al.	2016	Chinese	OR3A4	Up	Unknown	Plasma	0.852	Unclear	0.8694	0.9127	260	130	130	Healthy blood	7	[52]
Gao et al.	2016	Chinese	PVT1	Up	Intergenic	Plasma	0.797	Unclear	0.593	0.921	40	20	20	Healthy blood	6	[51]
Dastmalchi et al.	2021	Iranian	PVT1	Up	Intergenic	Tissue	0.71	Unclear	0.5588	0.7843	204	102	102	ANT	6	[37]
Yuan et al.	2016	Chinese	PVT1	Up	Intergenic	Tissue	0.728	Unclear	0.802	0.604	222	111	111	ANT	8	[38]
Shao et al.	2016	Chinese	RMRP	Up	Bidirectional	Plasma	0.639	Unclear	0.591	0.678	193	103	90	Healthy blood	6	[64]
Shao et al.	2016	Chinese	RMRP	Up	Bidirectional	Gastric juices	0.699	Unclear	0.564	0.754	84	39	45	Healthy juices	6	[64]
Sun et al.	2015	Chinese	RP11-119F7.4	Down	Unknown	Tissue	0.637	6.445	0.448	0.823	192	96	96	ANT	7	[39]
Mo et al.	2019	Chinese	RP11-555H23.1	Down	Unknown	Tissue	0.65	12.9	0.81	0.62	208	104	104	ANT	8	[40]
Jing et al.	2020	Chinese	RP11-731F5.2	Up	Unknown	Serum	0.78	Unclear	0.816	0.636	184	104	80	Healthy blood	6	[56]
Tang et al.	2020	Chinese	SGOL1-AS1	Up	Unknown	Tissue	0. 612	Unclear	0.877	0.4	130	65	65	ANT	6	[41]
Chen et al.	2016	Chinese	SNHG15	Up	Intronic	Tissue	0.722	4.43	0.642	0.745	212	106	106	ANT	8	[30]
Mei et al.	2013	Chinese	SUMO1P3	Up	Unknown	Tissue	0.666	2.31	0.659	0.636	192	96	96	ANT	7	[42]
Weiwei and Jianjun	2021	Chinese	TC0101441	Up	Unknown	Tissue	0.8082	8.45	0.817	0.923	318	159	159	ANT	6	[43]
Sun et al.	2015	Chinese	TINCR	Up	Intronic	Tissue	0.701	9.05	0.65	0.71	160	80	80	ANT	7	[39]
Li et al.	2021	Chinese	TUG1	Up	Intergenic	Serum	0.833	2.405	0.7419	0.8955	129	67	62	Healthy blood	9	[65]
Li et al.	2015	Chinese	UCA1	Up	Intergenic	Tissue	0.721	13.74	0.672	0.803	224	112	112	ANT	7	[57]
Gao et al.	2016	Chinese	UCA1	Up	Intergenic	Plasma	0.928	Unclear	0.892	0.803	40	20	20	Healthy blood	6	[51]
Ma et al.	2019	Chinese	ZEB1-AS1	Up	Antisense	Tissue	0.79	Unclear	0.821	0.792	143	84	59	ANT	7	[46]
Pan et al.	2017	Chinese	ZFAS1	Up	Antisense	Tissue	0.63	Unclear	0.5	0.787	188	94	94	ANT	8	[47]
Pan et al.	2017	Chinese	ZFAS1	Up	Antisense	Serum	0.792	Unclear	0.717	0.757	60	37	23	Healthy blood	6	[47]
Dastmalchi et al.	2021	Iranian	ZFAS1	Down	Antisense	Tissue	0.79	Unclear	0.6471	0.7941	204	102	102	ANT	6	[37]

**Table 2:** Diagnostic performance of lncRNAs.

Subgroups	Number	Pooled SEN (95% CI)	*I* ^2^ test (%) SEN	Pooled SPE (95% CI)	*I* ^2^ test (%) SPE	Pooled PLR (95% CI)	*I* ^2^ test (%) PLR	Pooled NLR (95% CI)	*I* ^2^ test (%) NLR	Pooled DOR (95% CI)	*I* ^2^ test (%) DOR	AUC (95% CI)	*Z*	*P* value
All studies	54	0.71 (0.67, 0.74)	84.23	0.76 (0.71, 0.79)	89.04	2.9 (2.5, 3.4)	83.95	0.39 (0.34, 0.43)	80.61	8 (6, 10)	78.9	0.79 (0.75, 0.82)		
*Location*														
Intergenic	22	0.72 (0.66, 0.77)	85.33	0.78 (0.72, 0.83)	88.42	3.2 (2.5, 4.1)	79.22	0.36 (0.30, 0.44)	83.17	9 (6, 13)	78.2	0.81 (0.78, 0.84)	-0.076	0.939
Antisense	11	0.73 (0.66, 0.79)	82.51	0.71 (0.61, 0.80)	90.08	2.6 (1.9, 3.4)	85.94	0.38 (0.30, 0.47)	74.65	7 (4, 10)	71	0.78 (0.75, 0.82)		
*Sample type*														
Circulating	19	0.76 (0.71, 0.81)	84.34	0.79 (0.71, 0.86)	92.98	3.7 (2.6, 5.3)	91.66	0.3 (0.24, 0.37)	83.61	12 (7, 20)	85	0.84 (0.80, 0.87)	-2.582	0.01
Tissue	32	0.69 (0.65, 0.73)	82.38	0.72 (0.68, 0.76)	83.41	2.5 (2.2, 2.9)	68.05	0.42 (0.38, 0.48)	75.4	6 (5, 7)	72.2	0.77 (0.73, 0.80)		
*Sample size*														
≤200	35	0.68 (0.63, 0.72)	79.78	0.75 (0.69, 0.79)	87.69	2.7 (2.2, 3.2)	77.63	0.43 (0.39, 0.48)	65.02	6 (5, 8)	57.3	0.76 (0.72, 0.80)	-1.259	0.208
>200	19	0.75 (0.70, 0.80)	87.58	0.77 (0.71, 0.82)	91.38	3.2 (2.5, 4.2)	87.41	0.33 (0.26, 0.41)	88.07	10 (6, 15)	88.2	0.82 (0.79, 0.86)		
*Quality*														
NOS score < 7	16	0.71 (0.65, 0.77)	81.76	0.75 (0.64, 0.83)	92.36	2.8 (2.0, 4.0)	87.66	0.38 (0.31, 0.47)	72.18	7 (5, 12)	80	0.78 (0.74, 0.82)	-0.303	0.762
NOS score ≥ 7	38	0.71 (0.66, 0.75)	85.42	0.76 (0.72, 0.80)	86.63	3.0 (2.5, 3.5)	77.38	0.39 (0.34, 0.44)	83.21	8 (6, 10)	78.9	0.80 (0.76, 0.83)		

*P* value: *Z*-test testing the between-subgroup AUC differences.

**Table 3:** Meta regression.

Var	Coeff.	Std. Err.	*P* value	RDOR	(95% CI)
(A) Four covariates (tau-squared estimate = 0.4232)					
Cte.	0.424	0.5777	0.4663	—	—
S	-0.288	0.0985	0.0053	—	—
Location	0.067	0.1223	0.589	1.07	(0.84, 1.37)
Sample type	0.588	0.2088	0.007	1.8	(1.18, 2.74)
Sample size	0.57	0.2185	0.012	1.77	(1.14, 2.74)
Quality	-0.058	0.2475	0.8166	0.94	(0.57, 1.55)
(B) Three covariates (tau-squared estimate = 0.4112)					
Cte.	0.333	0.4197	0.4313	—	—
S	-0.284	0.0965	0.005	—	—
Location	0.061	0.119	0.6097	1.06	(0.84, 1.35)
Sample type	0.594	0.2048	0.0056	1.81	(1.20, 2.73)
Sample size	0.562	0.2134	0.0112	1.75	(1.14, 2.69)
(C) Two covariates (tau-squared estimate = 0.4022)					
Cte.	0.362	0.4126	0.3845	—	—
S	-0.285	0.0957	0.0044	—	—
Sample type	0.615	0.1985	0.0032	1.85	(1.24, 2.76)
Sample size	0.564	0.2116	0.0104	1.76	(1.15, 2.69)
(D) One covariate (tau-squared estimate = 0.4752)					
Cte.	1.169	0.2957	0.0002	—	—
S	-0.271	0.1012	0.01	—	—
Sample type	0.599	0.2101	0.0063	1.82	(1.19, 2.78)
(E) One covariate (tau-squared estimate = 0.4775)					
Cte.	1.211	0.3318	0.0006	—	—
S	-0.199	0.097	0.0454	—	—
Sample size	0.534	0.2258	0.0218	1.71	(1.08, 2.68)

Var: variables; Cte: constant coefficient; S: statistic; RDOR: relative diagnostic odds ratio.

**Table 4:** The differential expression of lncRNAs in TCGA database.

Gene	Literature	TCGA	LogFC	*P* value	FDR	Reference
AC064834.1	Up	Up	2.829006	9.97*E*-08	8.19*E*-07	[31]
H19	Up	Up	2.271609	5.57*E*-08	4.82*E*-07	[25, 49, 53, 59, 62]
HOTAIR	Up	Up	5.542589	2.68*E*-43	4.93*E*-40	[9, 68]
HULC	Up	Up	2.062041	2.10*E*-05	0.0001057	[29, 55]
KRT18P55	Up	Up	1.660697	4.88*E*-11	7.46*E*-10	[27]
PVT1	Up	Up	1.626302	2.86*E*-19	1.79*E*-17	[38, 51]
UCA1	Up	Up	3.565784	7.92*E*-16	2.77*E*-14	[51, 57, 69]
C5orf66-AS1	Down	Down	-3.34947	7.57*E*-17	3.13*E*-15	[12]
LINC00086	Down	Down	-1.5301	9.53*E*-09	9.56*E*-08	[54]
ABHD11-AS1	Up	Down	-1.11544	5.92*E*-05	0.0002673	[22, 52]
GACAT2	Up	Down	-1.51797	9.65*E*-06	5.25*E*-05	[61]
LINC00982	Up	Down	-2.26334	2.59*E*-13	5.92*E*-12	[14]
RP11-731F5.2	Up	Down	-1.58792	0.000295	0.001116	[56]
TINCR	Up	Down	-2.73869	2.17*E*-12	4.17*E*-11	[39]
linc-ROR	Down	Up	1.882867	0.000465	0.001668	[35]
DLEU1	Up	No difference	0.582985	1.04*E*-06	6.94*E*-06	[32]
HEIH	Up	No difference	-0.74428	8.79*E*-11	1.29*E*-09	[33]
LINC00152	Up	No difference	0.971768	2.69*E*-09	3.00*E*-08	[10, 28, 30, 57]
SNHG15	Up	No difference	0.748521	3.62*E*-09	3.94*E*-08	[30]
SUMO1P3	Up	No difference	0.424479	0.0014487	0.0045057	[42]
TUG1	Up	No difference	0.461441	3.50*E*-08	3.16*E*-07	[65]
ZEB1-AS1	Up	No difference	0.386515	0.0063247	0.0160522	[46]
ZFAS1	Up	No difference	0.629700	7.56*E*-05	0.000333	[47]
BC002811	Up	Unclear				[60]
HIF1A-AS2	Up	Unclear				[39]
LINC01061	Up	Unclear				[58]
OR3A4	Up	Unclear				[52]
RMRP	Up	Unclear				[64]
SGOL1-AS1	Up	Unclear				[41]
TC0101441	Up	Unclear				[43]
AA174084	Down	Unclear				[21]
AC096655.1-002	Down	Unclear				[24]
AC138128.1	Down	Unclear				[23]
FER1L4	Down	Unclear				[23]
ncRuPAR	Down	Unclear				[36]
RP11-119F7.4	Down	Unclear				[39]
RP11-555H23.1	Down	Unclear				[40]

**Table 5:** Diagnostic efficacy of lncRNAs.

	AUC	(95% CI)	*P*	SEN	SPE	YI	Cut-off
lncRNA							
PVT1	0.949	(0.922, 0.976)	<0.01	0.808	0.969	0.777	0.8616
HOTAIR	0.942	(0.917, 0.967)	<0.01	0.891	0.906	0.797	0.0016
KRT18P55	0.890	(0.834, 0.947)	<0.01	0.765	0.906	0.671	0.0022
C5orf66-AS1	0.853	(0.790, 0.915)	<0.01	0.739	0.875	0.614	0.0218
UCA1	0.788	(0.713, 0.863)	<0.01	0.696	0.750	0.446	0.0855
AC064834.1	0.785	(0.720, 0.851)	<0.01	0.691	0.875	0.566	0.0001
HULC	0.754	(0.681, 0.827)	<0.01	0.608	0.844	0.452	0.0003
LINC00086	0.754	(0.678, 0.830)	<0.01	0.667	0.812	0.479	0.0867
H19	0.708	(0.610, 0.805)	<0.01	0.651	0.719	0.37	1.2771
Combined model							
PVT1+C5orf66-AS1	0.972	(0.951-0.992)	<0.01	0.941	0.937	0.878	0.7771

**Table 6:** Diagnostic efficacy of the combined model for GC with different stages.

Stage	SEN	SPE	YI	Cut-off	AUC	(95% CI)	*Z*	*P* value
I	0.902	0.937	0.840	0.4114	0.947	(0.875-0.984)		
II	0.929	0.938	0.866	0.5183	0.966	(0.922-0.989)	-0.703	0.482
III	0.973	0.938	0.910	0.4553	0.984	(0.952-0.997)	-1.504	0.133
IV	0.974	1	0.974	0.4548	0.998	(0.945-1.000)	-2.183	0.029
II+III+IV	0.953	0.938	0.891	0.6899	0.977	(0.955-0.991)	-1.192	0.233

*P* value: *Z*-test testing AUC differences of GC different stages which all compared to stage I.

## Data Availability

The data used to support the findings of this study are included within the article and supplementary information file.

## Abbreviations

ABHD11-AS1: Abhydrolase domain containing 11-antisense RNA1
ANT: Adjacent nontumor tissue
AUC: The area under the curve
CA19-9: Cancer antigen 19-9
CA72-4: Carbohydrate antigen 72-4
CEA: Carcinoembryonic antigen
CSCO: Chinese Society of Clinical Oncology
DOR: Diagnostic odds ratio
ESMO: European Society for Medical Oncology
GACAT2: Gastric cancer-associated transcript 2
GC: Gastric cancer
HOTAIR: HOX transcript antisense RNA
HULC: Highly upregulated in liver cancer
KRT18P55: Keratin 18 pseudogene 55
linc-ROR: Long intergenic nonprotein coding RNA, regulator of reprogramming
lncRNAs: Long noncoding RNAs
NATs: Natural antisense lncRNAs
NCCN: National Comprehensive Cancer Network
NLR: Negative likelihood ratio
NOS: Newcastle–Ottawa quality scale
PCA3: Prostate cancer-associated 3
PLR: Positive likelihood ratio
ROC: Receiver-operating characteristic
SEN: Sensitivity
SPE: Specificity
TCGA: The Cancer Genome Atlas
TSS: Transcription start site
UCA1: Urothelial cancer-associated 1.

## Supplementary Materials

Supplementary 1 supplement Table S1: the test of threshold effect and nonthreshold effect.

Supplementary 2 supplement Figure S1: sensitivity analysis of the pooled studies.

## References

[1] Quinn J. J., Chang H. Y. (2016). Unique features of long non-coding RNA biogenesis and function. *Nature Reviews Genetics*.

[2] Ponting C. P., Oliver P. L., Reik W. (2009). Evolution and functions of long noncoding RNAs. *Cell*.

[3] Huang B., Song J. H., Cheng Y. (2016). Long non-coding antisense RNA KRT7-AS is activated in gastric cancers and supports cancer cell progression by increasing KRT7 expression. *Oncogene*.

[4] Schalken J., Dijkstra S., Baskin-Bey E., van Oort I. (2014). Potential utility of cancer-specific biomarkers for assessing response to hormonal treatments in metastatic prostate cancer. *Therapeutic Advances in Urology*.

[5] Matouk I. J., Abbasi I., Hochberg A., Galun E., Dweik H., Akkawi M. (2009). Highly upregulated in liver cancer noncoding RNA is overexpressed in hepatic colorectal metastasis. *European Journal of Gastroenterology & Hepatology*.

[6] Sung H., Ferlay J., Siegel R. L. (2021). Global cancer statistics 2020: GLOBOCAN estimates of incidence and mortality worldwide for 36 cancers in 185 countries. *CA: a Cancer Journal for Clinicians*.

[7] Chen W., Zheng R., Baade P. D. (2016). Cancer statistics in China, 2015. *CA: a cancer journal for clinicians*.

[8] Wu M.-S., Wang H.-P., Lin C.-C. (1997). Loss of imprinting and overexpression of IGF2 gene in gastric adenocarcinoma. *Cancer Letters*.

[9] Xu Z., Chen H., Yang B., Liu X., Zhou X., Kong H. (2019). The association of HOTAIR with the diagnosis and prognosis of gastric cancer and its effect on the proliferation of gastric cancer cells. *Canadian Journal of Gastroenterology & Hepatology*.

[10] Shi Y., Sun H. (2020). Down-regulation of lncRNA LINC00152 suppresses gastric cancer cell migration and invasion through inhibition of the ERK/MAPK signaling pathway. *Oncotargets and Therapy*.

[11] Wang H. F., Lv J. Q., Li H. H., Wang W., Lin F. Q. (2020). High long non-coding LIFR-AS1 expression correlates with poor survival in gastric carcinoma. *European Review for Medical and Pharmacological Sciences*.

[12] Zhou Q., Li H., Jing J., Yuan Y., Sun L. (2020). Evaluation of C5orf66-AS1 as a potential biomarker for predicting early gastric cancer and its role in gastric carcinogenesis. *Oncotargets and Therapy*.

[13] Li S., Zhang M., Zhang H. (2020). Exosomal long noncoding RNA lnc-GNAQ-6:1 may serve as a diagnostic marker for gastric cancer. *Clinica chimica acta; international journal of clinical chemistry*.

[14] Fei Z. H., Yu X. J., Zhou M., Su H. F., Zheng Z., Xie C. Y. (2016). Upregulated expression of long non-coding RNA LINC00982 regulates cell proliferation and its clinical relevance in patients with gastric cancer. *Tumour biology: the journal of the International Society for Oncodevelopmental Biology and Medicine*.

[15] Zheng L., Cao J., Liu L. (2021). Long non-coding RNA LINC00982 up-regulates CTSF expression to inhibit gastric cancer progression via the transcription factor HEY1. *American journal of physiology Gastrointestinal and liver physiology*.

[16] Cui Z., Chen Y., Xiao Z. (2016). Long noncoding RNAs as auxiliary biomarkers for gastric cancer screening: a pooled analysis of individual studies. *Oncotarget*.

[17] Ding X., Wan X., Jiang H. (2015). The clinical value of ncRNAs in gastric cancer: a systematic review and meta-analyses. *Tumour biology : the journal of the International Society for Oncodevelopmental Biology and Medicine*.

[18] Moher D., Group P. R. I. S. M. A.-P., Shamseer L. (2015). Preferred reporting items for systematic review and meta-analysis protocols (PRISMA-P) 2015 statement. *Systematic Reviews*.

[19] Page M. J., McKenzie J. E., Bossuyt P. M. (2021). The PRISMA 2020 statement: an updated guideline for reporting systematic reviews. *PLoS Medicine*.

[20] Moher D., Liberati A., Tetzlaff J., Altman D. G., for the PRISMA Group (2009). Preferred reporting items for systematic reviews and meta-analyses: the PRISMA statement. *BMJ (Clinical research ed)*.

[21] Shao Y., Ye M., Jiang X. (2014). Gastric juice long noncoding RNA used as a tumor marker for screening gastric cancer. *Cancer*.

[22] Lin X., Yang M., Xia T., Guo J. (2014). Increased expression of long noncoding RNA ABHD11-AS1 in gastric cancer and its clinical significance. *Medical oncology (Northwood, London, England)*.

[23] Liu Z., Shao Y., Tan L., Shi H., Chen S., Guo J. (2014). Clinical significance of the low expression of FER1L4 in gastric cancer patients. *Tumour biology : the journal of the International Society for Oncodevelopmental Biology and Medicine*.

[24] Sun W., Wu Y., Yu X. (2013). Decreased expression of long noncoding RNA AC096655.1-002 in gastric cancer and its clinical significance. *Tumour biology : the journal of the International Society for Oncodevelopmental Biology and Medicine*.

[25] Chen J. S., Wang Y. F., Zhang X. Q. (2016). H19 serves as a diagnostic biomarker and up-regulation of H19 expression contributes to poor prognosis in patients with gastric cancer. *Neoplasma*.

[26] Chen W. M., Huang M. D., Kong R. (2015). Antisense long noncoding RNA HIF1A-AS2 is upregulated in gastric cancer and associated with poor prognosis. *Digestive Diseases and Sciences*.

[27] Wang Z., Ma B., Wang J. (2016). Upregulated long intergenic noncoding RNA KRT18P55 acts as a novel biomarker for the progression of intestinal-type gastric cancer. *Oncotargets and Therapy*.

[28] Pang Q., Ge J., Shao Y. (2014). Increased expression of long intergenic non-coding RNA LINC00152 in gastric cancer and its clinical significance. *Tumour biology : the journal of the International Society for Oncodevelopmental Biology and Medicine*.

[29] Zhao Y., Guo Q., Chen J. (2014). Role of long non-coding RNA HULC in cell proliferation, apoptosis and tumor metastasis of gastric cancer: a clinical and in vitro investigation. *Oncology Reports*.

[30] Chen W. M., Huang M. D., Sun D. P. (2016). Long intergenic non-coding RNA 00152 promotes tumor cell cycle progression by binding to EZH2 and repressing p15 and p21 in gastric cancer. *Oncotarget*.

[31] Abdi E., Latifi-Navid S., Zahri S., Kholghi-Oskooei V., Yazdanbod A. (2021). Novel long intergenic non-coding RNA--AC064834.1--misregulation in gastric cancer. *Gene Reports*.

[32] Ghodrati R., Safaralizadeh R., Dastmalchi N. (2021). Overexpression of lncRNA DLEU1 in gastric cancer tissues compared to adjacent non-tumor tissues. *Journal of Gastrointestinal Cancer*.

[33] Hui D., Fengjie L., Aichun J. (2021). Influences of lncRNA HEIH and DKK3 on the clinical features and prognosis of gastric cancer. *Journal of BUON : official journal of the Balkan Union of Oncology*.

[34] Tayefeh-Gholami S., Ghanbari M., Aghazadeh A. (2021). Prognostic value of lncRNA KRT18P55 in patients with intestinal type of gastric cancer. *Journal of Gastrointestinal Cancer*.

[35] Yu X., Ding H., Shi Y. (2020). Downregulated expression of linc-ROR in gastric cancer and its potential diagnostic and prognosis value. *Disease Markers*.

[36] Liu L., Yan B., Yang Z., Zhang X., Gu Q., Yue X. (2014). ncRuPAR inhibits gastric cancer progression by down-regulating protease-activated receptor-1. *Tumour biology : the journal of the International Society for Oncodevelopmental Biology and Medicine*.

[37] Dastmalchi N., Tayefeh-Gholami S., Rajabi A., Safaralizadeh R. (2021). PVT1 and ZFAS1 lncRNAs expressions and their biomarker value in gastric cancer tissue sampling among Iranian population. *Molecular Biology Reports*.

[38] Yuan C. L., Li H., Zhu L. (2016). Aberrant expression of long noncoding RNA PVT1 and its diagnostic and prognostic significance in patients with gastric cancer. *Neoplasma*.

[39] Sun J., Song Y., Chen X. (2015). Novel long non-coding RNA RP11-119F7.4 as a potential biomarker for the development and progression of gastric cancer. *Oncology Letters*.

[40] Mo X., Wu Y., Chen L. (2019). Global expression profiling of metabolic pathway-related lncRNAs in human gastric cancer and the identification of RP11-555H23.1 as a new diagnostic biomarker. *Journal of Clinical Laboratory Analysis*.

[41] Tang L. H., Ma Z., He Z., Tan L., Xiao J. W. (2020). Expression and clinical significance of long non-coding RNA SGOL1-AS1 in gastric cancer. *Sichuan Medical Journal*.

[42] Mei D., Song H., Wang K. (2013). Up-regulation of SUMO1 pseudogene 3 (SUMO1P3) in gastric cancer and its clinical association. *Medical oncology (Northwood, London, England)*.

[43] Weiwei W., Jianjun W. (2021). Identification of long noncoding RNA TC0101441 as a novel biomarker for diagnosis and prognosis of gastric cancer. *International Journal of Clinical and Experimental Pathology*.

[44] Xu T. P., Liu X. X., Xia R. (2015). SP1-induced upregulation of the long noncoding RNA TINCR regulates cell proliferation and apoptosis by affecting KLF2 mRNA stability in gastric cancer. *Oncogene*.

[45] Zheng Q., Wu F., Dai W. Y. (2015). Aberrant expression of UCA1 in gastric cancer and its clinical significance. *Clinical & translational oncology : official publication of the Federation of Spanish Oncology Societies and of the National Cancer Institute of Mexico*.

[46] Ma M. H., An J. X., Zhang C. (2019). ZEB1-AS1 initiates a miRNA-mediated ceRNA network to facilitate gastric cancer progression. *Cancer Cell International*.

[47] Pan L., Liang W., Fu M. (2017). Exosomes-mediated transfer of long noncoding RNA ZFAS1 promotes gastric cancer progression. *Journal of Cancer Research and Clinical Oncology*.

[48] Chen X., Sun J., Song Y. (2014). The novel long noncoding RNA AC138128.1 may be a predictive biomarker in gastric cancer. *Medical Oncology (Northwood, London, England)*.

[49] Arita T., Ichikawa D., Konishi H. (2013). Circulating long non-coding RNAs in plasma of patients with gastric cancer. *Anticancer Research*.

[50] Cai C., Zhang H., Zhu Y. (2019). Serum exosomal long noncoding RNA pcsk2-2:1 as a potential novel diagnostic biomarker for gastric cancer. *Oncotargets and Therapy*.

[51] Gao J., Cao R., Mu H. (2015). Long non-coding RNA UCA1 may be a novel diagnostic and predictive biomarker in plasma for early gastric cancer. *International Journal of Clinical and Experimental Pathology*.

[52] Guo X., Yang Z., Zhi Q. (2016). Long noncoding RNA OR3A4 promotes metastasis and tumorigenicity in gastric cancer. *Oncotarget*.

[53] Hashad D., Elbanna A., Ibrahim A., Khedr G. (2016). Evaluation of the role of circulating long non-coding RNA H19 as a promising novel biomarker in plasma of patients with gastric cancer. *Journal of Clinical Laboratory Analysis*.

[54] Ji B., Huang Y., Gu T., Zhang L.’., Li G., Zhang C. (2019). Potential diagnostic and prognostic value of plasma long noncoding RNA LINC00086 and miR-214 expression in gastric cancer. *Cancer biomarkers : section A of Disease markers*.

[55] Jin C., Shi W., Wang F. (2016). Long non-coding RNA HULC as a novel serum biomarker for diagnosis and prognosis prediction of gastric cancer. *Oncotarget*.

[56] Jing R., Liu S., Jiang Y., Zong W., Ju S., Cui M. (2020). Determination of serum RP11-731F5.2 as a noninvasive biomarker for gastric cancer diagnosis and prognosis. *Pathology Research and Practice*.

[57] Li Q., Shao Y., Zhang X. (2015). Plasma long noncoding RNA protected by exosomes as a potential stable biomarker for gastric cancer. *Tumour biology : the journal of the International Society for Oncodevelopmental Biology and Medicine*.

[58] Liang W., Xia B., Yan M., Zhai G., Li M. (2021). Enhanced LINC01061 levels as a serum biomarker in gastric cancer and promotion of malignant transformation. *Oncology Research and Treatment*.

[59] Mohamed W. A., Schaalan M. F., Ramadan B. (2019). The expression profiling of circulating miR-204, miR-182, and lncRNA H19 as novel potential biomarkers for the progression of peptic ulcer to gastric cancer. *Journal of Cellular Biochemistry*.

[60] Zhou F. Y., Guo J. C., Xie P., Zhang Z. L. (2020). Diagnostic value of lncRNA BC002811 combined with serum tumor markers CA125 and CA199 in gastric cancer. *Chinese Journal of Public Health Engineering*.

[61] Tan L., Yang Y., Shao Y., Zhang H., Guo J. (2016). Plasma lncRNA-GACAT2 is a valuable marker for the screening of gastric cancer. *Oncology Letters*.

[62] Zhou X., Yin C., Dang Y., Ye F., Zhang G. (2015). Identification of the long non-coding RNA H19 in plasma as a novel biomarker for diagnosis of gastric cancer. *Scientific Reports*.

[63] Yu J. R., Fang C., Zhang Z. Y. (2020). H19 rises in gastric cancer and exerts a tumor-promoting function via miR-138/E2F2 axis. *Cancer Management and Research*.

[64] Shao Y., Ye M., Li Q. (2016). lncRNA-RMRP promotes carcinogenesis by acting as a miR-206 sponge and is used as a novel biomarker for gastric cancer. *Oncotarget*.

[65] Li D., Sun M., Ma C. (2021). lncRNA TUG1 can inhibit the proliferation and invasion of gastric cancer by increasing miR-506 expression. *Acta Medica Mediterranea*.

[66] Yang Y., Shao Y., Zhu M. (2016). Using gastric juice lncRNA-ABHD11-AS1 as a novel type of biomarker in the screening of gastric cancer. *Tumour biology : the journal of the International Society for Oncodevelopmental Biology and Medicine*.

[67] Schaalan M., Mohamed W., Fathy S. (2020). MiRNA-200c, MiRNA-139 and ln RNA H19; new predictors of treatment response in H-pylori- induced gastric ulcer or progression to gastric cancer. *Microbial Pathogenesis*.

[68] Xiangyang Z., Chengqiang Y., Yongqiang S., Gastroenterology D. O. (2017). Expression and significance of long non-coding RNA HOTAIR in gastric cancer. *Jiangsu Medical Journal*.

[69] Ke D., Li H., Zhang Y. (2017). The combination of circulating long noncoding RNAs AK001058, INHBA-AS1, MIR4435-2HG, and CEBPA-AS1 fragments in plasma serve as diagnostic markers for gastric cancer. *Oncotarget*.

[70] Jin Z., Jiang W., Wang L. (2015). Biomarkers for gastric cancer: progression in early diagnosis and prognosis (review). *Oncology Letters*.

[71] Qi P., Du X. (2013). The long non-coding RNAs, a new cancer diagnostic and therapeutic gold mine. *Inc*.

[72] Derrien T., Johnson R., Bussotti G. (2012). The GENCODE v7 catalog of human long noncoding RNAs: analysis of their gene structure, evolution, and expression. *Genome Research*.

[73] Yarmishyn A. A., Kurochkin I. V. (2015). Long noncoding RNAs: a potential novel class of cancer biomarkers. *Frontiers in Genetics*.

[74] Gharib E., Anaraki F., Baghdar K. (2019). Investigating the diagnostic performance of HOTTIP, PVT1, and UCA1 long noncoding RNAs as a predictive panel for the screening of colorectal cancer patients with lymph node metastasis. *Journal of Cellular Biochemistry*.

[75] Zhou D. D., Liu X. F., Lu C. W., Pant O. P., Liu X. D. (2017). Long non-coding RNA PVT1: emerging biomarker in digestive system cancer. *Cell Proliferation*.

[76] Rui X., Xu Y., Jiang X., Ye W., Huang Y., Jiang J. (2018). Long non-coding RNA C5orf66-AS1 promotes cell proliferation in cervical cancer by targeting miR-637/RING1 axis. *Cell Death & Disease*.

[77] Luo W., Wang M., Liu J., Cui X., Wang H. (2020). Identification of a six lncRNAs signature as novel diagnostic biomarkers for cervical cancer. *Journal of Cellular Physiology*.

[78] Guo W., Lv P., Liu S. (2018). Aberrant methylation-mediated downregulation of long noncoding RNA C5orf66-AS1 promotes the development of gastric cardia adenocarcinoma. *Molecular Carcinogenesis*.

