# Supplementary material for: Abnormally Expressed lncRNAs as Potential Biomarkers for Gastric Cancer Risk: A Diagnostic Meta-Bioinformatics Analysis
Source: Biomed Res Int. 2022 Nov 4;2022:6712625. doi: 10.1155/2022/6712625 (PMC9652703; doi:10.1155/2022/6712625)
Supplement: Supplementary 1 — supplement Table S1: the test of threshold effect and nonthreshold effect. [file 6712625.f1.docx]

**Supplement TableS1 The test of threshold effect and non-threshold effect**

| Analysis | Spearman correlation | *P* value | Cochran’s-Q test | *P* value | Heterogeneity | |
| --- | --- | --- | --- | --- | --- | --- |
|  | Coefficient |  |  |  | Threshold effect | Non-threshold effect |
| Overall(54) | 0.25 | 0.069 | 251.75 | <0.001 | NO | YES |
