# Supplementary figures and images for: Abnormally Expressed lncRNAs as Potential Biomarkers for Gastric Cancer Risk: A Diagnostic Meta-Bioinformatics Analysis
Source: Biomed Res Int. 2022 Nov 4;2022:6712625. doi: 10.1155/2022/6712625 (PMC9652703; doi:10.1155/2022/6712625)

# Meta-analysis estimates, given named study is omitted

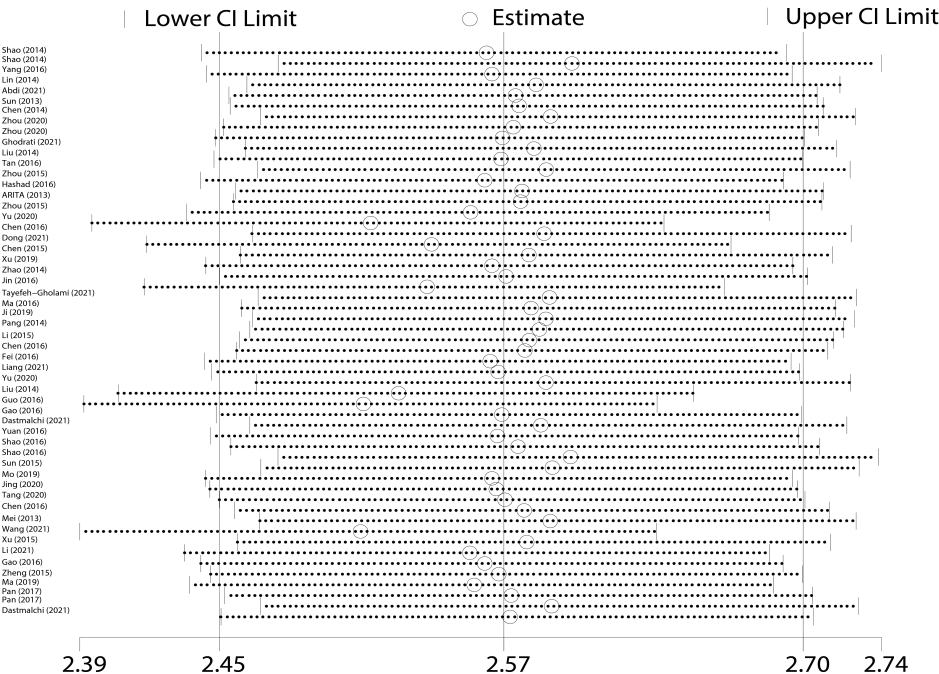

Supplement: Supplementary 2 — supplement Figure S1: sensitivity analysis of the pooled studies. [file 6712625.f2.pdf]
